# Comparing Web-Based Mindfulness With Loving-Kindness and Compassion Training for Promoting Well-Being in Pregnancy: Protocol for a Three-Arm Pilot Randomized Controlled Trial

**DOI:** 10.2196/19803

**Published:** 2020-10-14

**Authors:** Amy Louise Finlay-Jones, Jacqueline Ann Davis, Amanda O'Donovan, Keerthi Kottampally, Rebecca Anne Ashley, Desiree Silva, Jeneva Lee Ohan, Susan L Prescott, Jenny Downs

**Affiliations:** 1 Telethon Kids Institute Nedlands Australia; 2 Curtin University Bentley Australia; 3 University of Western Australia Crawley Australia; 4 Murdoch University Murdoch Australia; 5 Edith Cowan University Joondalup Australia; 6 Joondalup Health Campus Joondalup Australia; 7 Perth Children's Hospital Nedlands Australia

**Keywords:** mindfulness, compassion, pregnancy, telemedicine, mental health, public health, intervention

## Abstract

**Background:**

Promoting psychological well-being and preventing distress among pregnant women is an important public health goal. In addition to adversely impacting the mother’s health and well-being, psychological distress in pregnancy increases the risk of poor pregnancy outcomes, compromises infant socioemotional development and bonding, and heightens maternal and child vulnerability in the postpartum period. Mindfulness and compassion-based interventions show potential for prevention and early intervention for perinatal distress. As there is an established need for accessible, scalable, flexible, and low-cost interventions, there is increased interest in the delivery of these programs on the web. This project aims to pilot a three-arm randomized controlled trial (RCT) to determine the feasibility of a full-scale RCT comparing 2 web-based interventions (mindfulness vs loving-kindness and compassion) with a web-based active control condition (progressive muscle relaxation).

**Objective:**

The primary objective of this study is to assess the feasibility of an RCT protocol comparing the 3 conditions delivered on the web as a series of instructional materials and brief daily practices over a course of 8 weeks. The second objective is to explore the experiences of women in the different intervention conditions. The third objective is to estimate SD values for the outcome measures to inform the design of an adequately powered trial to determine the comparative efficacy of the different conditions.

**Methods:**

Pregnant women (n=75) participating in a longitudinal birth cohort study (the ORIGINS project) will be recruited to this study from 18 weeks of gestational age. We will assess the acceptability and feasibility of recruitment and retention strategies and the participants’ engagement and adherence to the interventions. We will also assess the experiences of women in each of the 3 intervention conditions by measuring weekly changes in their well-being and engagement with the program and by conducting a qualitative analysis of postprogram interviews.

**Results:**

This project was funded in September 2019 and received ethics approval on July 8, 2020. Enrollment to the study will commence in September 2020. Feasibility of a full-scale RCT will be assessed using ADePT (*a process for decision making after pilot and feasibility trials*) criteria.

**Conclusions:**

If the study is shown to be feasible, results will be used to inform future full-scale RCTs. Evidence for flexible, scalable, and low-cost interventions could inform population health strategies to promote well-being and reduce psychological distress among pregnant women.

**Trial Registration:**

Australian New Zealand Clinical Trials Registry Number 12620000672954p; http://anzctr.org.au/ACTRN12620000672954p.aspx

**International Registered Report Identifier (IRRID):**

PRR1-10.2196/19803

## Introduction

### Background

Maternal mental health in pregnancy is an issue of substantial public health importance [1]. Anxiety and depression are the most common postpartum difficulties experienced by women globally [2], with an average prevalence of 10% to 15%, although rates as high as 64% have been reported in US samples [2]. Psychological distress during pregnancy is associated with adverse impacts for both the mother and child [3-6]. For example, prenatal symptoms of depression and anxiety are associated with impaired fetal growth [7], risk of child emotional and behavioral problems [8-13], and differences in child brain morphology [14]. These findings align with the Developmental Origins of Health and Disease model [15], which posits that the characteristics of the perinatal environment exert lifelong influences on offspring health risk and resilience. Maternal mental health difficulties in pregnancy can also increase the risk of adjustment difficulties in the postnatal period [16,17].

Despite the availability of accessible and effective treatments for mood disorders, approximately half of the women experiencing perinatal distress do not seek help [18-20]. Such findings demonstrate a need for universal mental health promotion efforts that complement targeted treatment programs and span prevention through early intervention. Nonpharmacological approaches to mood management are also preferred by pregnant women because of concerns about the effects of pharmacological interventions on the developing fetus [21]. Furthermore, research on mental health risk and resilience also illustrates the dual benefits of targeting subclinical symptoms of distress and increasing positive mental health and well-being. For example, although a history of psychiatric symptoms predisposes individuals to future psychopathology, positive mental health both supports recovery from distress and protects against poor mental health in the future [22-25]. Studies with pregnant women have found that prenatal positive mental health traits such as positive affect and optimism are protective of postpartum depression [26,27] and birth outcomes such as preterm delivery [28]. A dual approach aligns with two-factor models of mental health, which recognize distress and well-being as 2 distinct but interrelated continua [22,29,30].

### Meditation-Based Interventions

Meditation-based interventions, including mindfulness-based interventions (MBIs) and compassion-based interventions (CBIs), are promising approaches for improving mental health and reducing psychological distress in the perinatal period. There is considerable evidence demonstrating the effectiveness of MBIs in reducing symptoms of psychological distress and promoting positive mental health in community populations [31,32] and clinical and at-risk groups [33,34]. CBIs (used here to denote meditation interventions focused on loving-kindness, self-compassion, and compassion for others) are similar to MBIs in that standardized protocols tend to be delivered over 8 weeks, with meditation practice considered an *active ingredient* in intervention effectiveness [35,36]. However, there are also important differences: although MBIs focus on cultivating nonjudgmental awareness and acceptance of all present-moment experiences [37], CBIs involve the intentional cultivation of positive affective states (eg, love, kindness, compassion, joy) [38].

Although the literature on CBIs is sparse relative to that for MBIs, reviews and meta-analyses also document positive effects of compassion and loving-kindness meditation practices on positive mental health [39] and psychological distress [35,40,41]. More recent work has demonstrated the greater efficacy of CBI protocols over MBI protocols for some outcomes. For example, Le Nguyen et al [42] reported the superior effects of loving-kindness training over mindfulness training for improving telomere length in novice meditators. Furthermore, Trautwein et al [37] demonstrated the differential benefits of different types of meditation training (attention and loving-kindness) on various cognitive and affective outcomes. Although these findings provide the basis for the development of more targeted intervention approaches, it is not clear which of these outcomes is most relevant for promoting mental health and well-being in pregnant women.

### MBIs and CBIs in Pregnancy

Evidence drawn from observational and experimental studies illustrates the potential benefit of MBIs and CBIs in reducing distress and promoting mental health in the perinatal period. For example, longitudinal studies have found significant inverse associations between trait mindfulness during pregnancy and both depressed mood across pregnancy and low birth weight (<10th percentile, after adjusting for gestational age, parity, and sex) [43]. Preliminary evidence also indicates that maternal mindfulness during pregnancy is associated with better behavioral and regulatory outcomes in infants and children [44,45]. However, results from systematic reviews of perinatal MBIs have been variable [46-48]. Although the literature suggests that mindfulness training in the perinatal period reduces perinatal stress and anxiety [48], there is mixed evidence regarding reductions in depression [48]. This is partly linked to heterogeneity in sample types and methodology as well as differences across intervention protocols.

Although initial evidence demonstrates associations among maternal self-compassion, well-being, and adjustment [49-51], there are only very few studies assessing CBIs in the perinatal period. Those that are available have reported encouraging results. For example, a proof-of-concept study compared a 2-week web-based CBI with web-based cognitive behavioral therapy (CBT) for perinatal women and those planning pregnancy (n=123) [52]. Participants in the 2 groups had similar outcomes in terms of affect, self-reassurance, self-criticism, and self-compassion. However, CBI was superior to CBT in terms of reducing symptoms of depression and anxiety. In another study, a 6-session, 3-week loving-kindness and compassion group program was compared with pregnancy yoga and an untreated control group in a sample of pregnant women (n=109) [53]. In this study, participants in the intervention group reported significant improvements in maternal-fetal attachment, mindfulness, and positive emotion relative to the control groups at posttest and follow-up. There are currently no head-to-head studies comparing the efficacy of MBIs and CBIs among pregnant women.

### Web-Based Meditation-Based Interventions

There are a number of potential benefits of delivering MBIs and CBIs via the internet [52,54], such as the capacity to implement across populations, to reach individuals who might struggle to access face-to-face services, the potential to improve cost-efficiencies, and the ability to provide greater flexibility to service users [55]. Meta-analyses have found small-to-moderate effect sizes for web-based and self-guided MBIs in promoting well-being and reducing symptoms of psychological distress [56]. Although there are limited studies of internet-delivered CBIs, 1 RCT of web-based compassion training for self-critical individuals reported moderate-to-large effect sizes for reductions in distress, relative to usual care [57]. However, it is currently unclear whether MBIs and CBIs delivered on the web are a feasible and efficacious means of reducing stress and promoting well-being in the perinatal period [58]. Available evidence for internet-delivered MBIs highlights some issues with intervention feasibility. For example, 1 RCT found evidence that compared with waitlist control, a web-based, 8-week self-guided MBI was associated with significant reductions in stress and pregnancy distress among pregnant women who completed the intervention; however, attrition exceeded 50% [59]. In the study by Kelman et al [52] on web-based CBI versus web-based CBT for perinatal women and those planning pregnancy, retention at a 2-week follow-up was 62% for CBI versus 71% for CBT.

As the field of pre- and perinatal meditation training advances, questions are raised about the types of meditation training, their suitability, and expected outcomes. In addition, there are enduring questions about how best to engage pregnant women in internet-delivered mental health interventions. Initial findings regarding engagement and retention within studies suggest a need for attention to consumer-focused design in the developmental stage of the intervention protocols [60]. This pilot study is intended as a precursor to a larger study (the Mums’ Minds Matter [MMM] study), in which we aim to address some of these issues. The MMM study will involve a three-arm RCT comparing web-based mindfulness training, web-based loving-kindness and compassion training (LKCT), and web-based progressive muscle relaxation (PMR; active control) to improve positive mental health and psychological distress among pregnant women. Both experimental conditions in the study were developed with input from consumers (ie, women who had been pregnant) and were designed in line with the instructional design framework for MBIs proposed by Lippmann et al [54]. PMR was selected as an active control condition as it is considered safe for pregnant women, is associated with physical and psychological benefits in the perinatal period, and could be matched to the format and duration of the experimental conditions [61].

### Objectives

This study aims to pilot the overall MMM study design, including the recruitment, screening, randomization, assessment, and intervention methods to be used in the full-scale trial. In addition to piloting these methods, this study has 3 objectives: (1) to assess the feasibility of the full-scale trial by measuring recruitment to the trial and retention, engagement, and completion rates for each arm of the trial; (2) to explore the experiences of women in the study; and (3) to estimate the standard deviation values for each of the outcome measures to inform sample size calculations for the main trial.

## Methods

### Study Design

**Figure 1:**
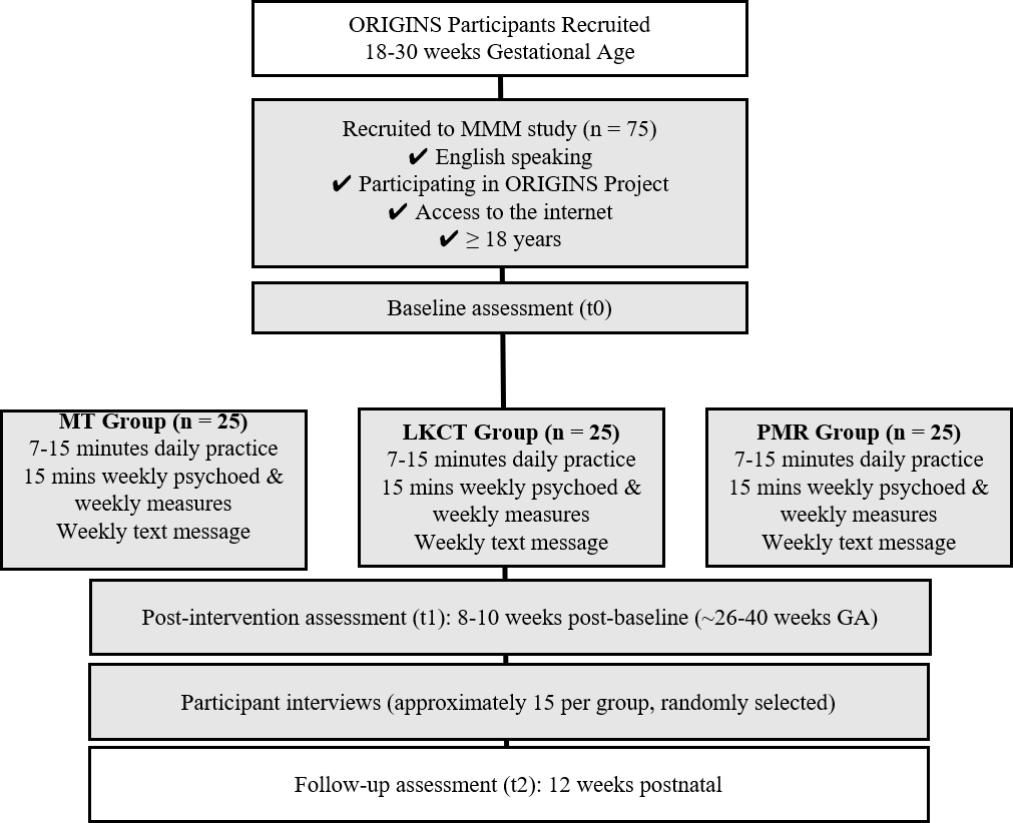
Participant flow through the study.

This is a randomized controlled trial (RCT) with 3 parallel groups: (1) mindfulness training, (2) LKCT, and (3) PMR and a repeated measure design. The trial is single blinded, with block randomization to have equal allocation of participants to groups stratified by parity. The trial is registered with the Australian New Zealand Clinical Trials Registry; the trial registration number of the study is 12620000672954p. This study was approved by the Joondalup Health Campus (JHC) Human Research Ethics Committee. An overview of the study design is shown in Figure 1.

### Trial Site and Participating Centers

The MMM interventions are all web-based, and all data will be collected electronically. As the study will recruit women receiving antenatal care at JHC, this health campus is considered the physical trial site.

### Participants

#### Eligibility Criteria

Eligible participants are pregnant women participating in the ORIGINS project (ORIGINS) and receiving antenatal care at a metropolitan health campus (JHC). ORIGINS is a longitudinal birth cohort study that recruits pregnant women and their partners from 12 weeks of gestational age and follows them and their child until 5 years postpartum. ORIGINS is open to any women who are birthing their baby at JHC [62]. Participants considered for inclusion in the MMM study will be women (1) at 18 to 30 weeks of pregnancy; (2) aged ≥18 years; (3) able to read, write, and understand English; and (4) able to access the internet.

#### Sample Size

We aim to recruit 25 participants per treatment arm (75 participants in total). The recommended sample sizes per treatment arm for pilot trials are between 15 and 25 when the standardized effect sizes for outcomes in the main trial are expected to be medium (0.5) to small (0.2) [63].

### Recruitment

Participants will be recruited at or following their first antenatal assessment for ORIGINS. Participants will receive recruitment materials (a recruitment video and website link) via email and/or text message. Interested participants will be directed to the study website to complete electronic consent forms and web-based screening measures via REDCap (Vanderbilt University). In addition to assessing eligibility criteria and parity (for stratification), the screening assessment will include the Edinburgh Postnatal Depression Scale (EPDS). This is a valid tool for detecting symptoms of depression during pregnancy and postnatally [64]. Participants scoring above the thresholds for the risk of depression (total score ≥13) or reporting thoughts of self-harm (score of 1-3 on item 10) will receive a phone call from a registered psychologist to discuss referral to more targeted support services. They will not be excluded from participation in the study.

### Randomization, Allocation, and Masking of the Intervention Conditions

Following screening, eligible participants will be asked to complete a web-based assessment battery comprising the outcome measures for the main trial. Upon completion of this assessment battery, the REDCap randomization module will be used to randomize participants to 1 of the 3 conditions. Randomization will be stratified by parity (nulliparous or not). The research assistant (RA) will be notified by email when a participant has been randomized. Although participants will not be blinded to the intervention condition, the RA will code participants’ condition allocation in the database so that the data analyst is blind to allocation. Minimal sufficient identifying data will be used to link MMM participant IDs to participant IDs in the ORIGINS study. This will be used to obtain the ORIGINS study data on participants’ sociodemographic and birth outcome data.

### Participant Procedure

Once a participant has been randomized, the RA will add the participant to their allocated intervention condition in Teachable, the web-based system used to deliver the programs. Participants will then receive an automated email from Teachable prompting them to create a password. Participants will also receive a phone call from the RA to confirm that they have successfully logged in to the program and to answer any participant questions. Participants will be asked to nominate a start date for the program within the next week.

Participants will access their allocated condition within the MMM program either on a computer (via the Teachable website) or on a smart device via the Teachable app. On the basis of their nominated start date, participants will receive an automated weekly text message with a link to the weekly outcome measures. These measures involve self-reports of stress, affect, and emotion regulation and are also designed to prompt self-reflection. There are also questions about daily practice, intentions to continue practice, and barriers and facilitators to practice. Participants who indicate that they do not intend to continue practice in the next week will be asked if they wish to opt out of the program. Participants choosing to opt out will be directed to a survey, which will ask them about the reasons for opting out of the program and request program feedback. At the conclusion of the 8-week intervention period, participants will be emailed a link to the t1 (posttest) assessment battery. Participants will be emailed again with a link to the t2 (follow-up) assessment battery 3 months after giving birth.

### Interventions

All intervention conditions will be delivered on the web using the Teachable platform. The program is structured so that each day of the intervention is delivered as a separate *lesson*. The same intervention structure will be used for each condition: 8 weeks, with 1 formal practice (audio recording) to be practiced daily each week. Participants will also receive text instructions for 1 informal practice each week, which they are encouraged to practice as often as feasible. The informal practices are designed to support participants to integrate skills and learning into their daily activities.

Both the mindfulness training and LKCT interventions were developed following a two-step process. First, we conducted a scoping review of the literature to determine key components of MBIs and CBIs administered during the perinatal period. In addition to extracting data regarding intervention characteristics (eg, weekly themes, duration, mode of delivery, home practice), we also extracted data regarding specific considerations for delivering meditation-based interventions during pregnancy. These data were used to develop a draft intervention protocol for the mindfulness training and LKCT conditions. The protocol was developed by AF and AO, who have masters-level training in clinical psychology and are both certified teachers of MBIs and CBIs. The PMR condition was based on exercises described by Jacobson [65] and adapted for pregnancy by excluding muscle tension in the abdominal area.

The intervention protocol was also informed by prior theoretical and empirical work describing the proposed mechanisms by which mindfulness and loving-kindness and compassion training impacts positive mental health and distress. For example, dismantling studies have supported the theory that acceptance is a necessary component for MBIs to improve positive emotions [66], whereas perspectives and empirical data on loving-kindness and compassion meditation highlight the central role of self-soothing, caring motivations, and socioaffective processes (eg, directing loving thoughts toward another) [37,67-69]. Finally, the structure of the intervention conditions was informed by the instructional design model for internet-based MBIs proposed by Lippmann et al [54]. These include the use of formal and informal practices, provision of educational and supportive material, and the use of reminders (via text message) at least once a week. The core themes and practices for each condition are described in Table 1. Further information on the formal practices can be found in Multimedia Appendix 1.

**Table 1:** Outline of key themes and practices for each condition.

Components	Mindfulness training	Loving-kindness and compassion training	Progressive Muscle Relaxation
Themes	Nonjudgmental awareness of present-moment experiences (thoughts, feelings, and sensations); acceptance of present-moment experiences (thoughts, feelings, and sensations); using the breath as an anchor for attention; and how mindfulness can support perinatal health	Friendliness toward self; loving-kindness toward self, baby, and others; self-compassion; responding to difficulties with kindness and compassion; and how loving-kindness and compassion can support perinatal health	Relaxation and how relaxation can support perinatal health
Formal practices	Body scan, breath-focused meditation, walking meditation, and mountain meditation	Compassionate check-in, compassionate body scan, soothing image practice, loving-kindness practice, and responding to emotions with compassion	Progressive muscle relaxation exercise
Informal practice	Mindfulness of daily activities (chores, showering, and waking up), mindful communication, mindful walking in nature, and 3-min breathing space	Body softening practice, micromoments of soothing, on-the-spot loving kindness practice, and savoring practice	Informal relaxation applied in different contexts (eg, going to bed, driving, waking up)

Consumer testing and input for the mindfulness training and LKCT protocols were sought from a group of 16 women (8 per condition) who had a recent experience of pregnancy and had given birth in the past 5 years. Women accessed the meditation recordings and associated content on the web via the Teachable platform over the course of 4 weeks. They were asked to practice formal and informal practices and provide feedback on each practice using a web-based questionnaire. Reponses were collated and discussed in a series of focus groups (2 per treatment condition) where participants described their motivations and experiences with the intervention protocol and suggested revisions to the content and structure. For example, based on participant feedback, the practices in weeks 1 and 2 for each condition were changed from 15 min to 7 min and 10 min, respectively. Recordings were also amended to include background music. LKCT meditation practices were also slightly modified with regard to the terms used (eg, removal of the word *suffering*). The resulting intervention protocols involved daily practice of 7 min in week 1, 10 min in week 2, and 15 min in weeks 3 to 8, coupled with psychoeducation and 1 informal practice per week.

### Data Collection

#### Study Outcomes

We will assess several metrics to determine the feasibility of the study across the following domains: recruitment, randomization, retention, and adherence (Table 2). To assess the feasibility of the assessment battery and calculate standard deviations on the measures, the main outcome measures for the full-scale RCT will be used (Table 3). Postintervention interviews will be conducted with a randomly selected sample of participants (n=approximately 15 in each condition) to gather further data on engagement with practice, reasons for attrition, satisfaction with the program, enjoyment and value of the program, and barriers and facilitators to engagement. Participants will be invited to partake in a semistructured interview by telephone, videoconference, or at the JHC clinic. Table 4 shows the Standard Protocol Items Recommendations for Interventional Trials (SPIRIT) schedule of enrollment, interventions, and assessment.

**Table 2:** Summary of outcomes and metrics for pilot study.

Outcome	Metrics
Recruitment	Number of participants screened per week Number of participants enrolled per week Proportion of recruited ORIGINS participants electing to enroll
Randomization	Proportion of eligible participants who access the web-based intervention following randomization Proportion of eligible participants who complete at least one session (one day) of the web-based intervention
Retention	Proportion of participants that complete t0 (baseline) measures after commencing them Condition-specific completion rates following randomization (completion defined as ≥50% of modules accessed at least once) Proportion of participants commencing baseline measures that complete time 1 (posttest) and time 2 (follow-up) measures
Adherence	Self-reported completion of practice Lesson completions in Teachable program
Acceptability	Proportion of participant opt-outs from each intervention condition Proportion of participant withdrawals from the study Self-reported intervention and study satisfaction

**Table 3:** Summary of outcomes and measures for a full-scale randomized controlled trial.

Outcomes		Measures
**Primary outcome**		
	Mental well-being	Mental Health Continuum–Short Form (14 items) [70]
**Secondary outcomes**		
	Depression	Edinburgh Postnatal Depression Scale (10 items) [71]
	Anxiety	Generalized Anxiety Disorder (7 items) [72]
	Stress	Perceived Stress Scale (10 items) [73]
	Self-compassion	Self-Compassion Scale–Short Form (12 items) [74]
	Mindfulness	Mindful Attention and Awareness Scale (15 items) [75]
	Emotion regulation	Difficulties in Emotion Regulation Scale (16 items) [76]
	Affect	Positive and Negative Affect Scale–Short Form (10 items) [77]
	Quality of life	EuroQol–5 Dimension (5 items) [78]
**Tertiary outcomes**		
	Program satisfaction	Likert scale developed for the study to assess satisfaction with the assigned condition and the overall study (11 items)
	Program engagement	Likert scale developed for the study to assess completion of daily practice, completion barriers and facilitators, and intention to complete practice in the following week (6 items)

**Table 4:** Standard Protocol Items Recommendations for Interventional Trials (SPIRIT) schedule of enrollment, interventions, and assessment.

Study activities in each phase		Timepoint				
		Enrollment	Postallocation			
		Week 1 (t0)	Week 2	Week 9	Week 10 (t1)	3 months postpartum (t2)
**Preintervention**						
	Eligibility screening	X^a^	N/A^b^	N/A	N/A	N/A
	Informed consent	X	N/A	N/A	N/A	N/A
	Allocation	X	N/A	N/A	N/A	N/A
**Intervention**						
	Web-based mindfulness training	N/A	X	X	N/A	N/A
	Web-based loving-kindness and compassion training	N/A	X	X	N/A	N/A
	Web-based progress muscle relaxation	N/A	X	X	N/A	N/A
**Assessments**						
	Demographics	X	N/A	N/A	N/A	N/A
	Self-reported mental well-being, depression, anxiety, self-compassion, mindfulness, and quality of life	X	N/A	N/A	X	X
	Self-reported affect, stress, and emotion regulation	X	N/A	N/A	X	X
	Program engagement	N/A	X	X	N/A	N/A
	Interviews	N/A	N/A	N/A	X	X

^a^X denotes when the study activity occurs.

^b^N/A: not applicable.

#### Data Management

On enrollment, all participants will be given a unique identifier that will be used in all database records. Although patient identifying information (first name and initials, date of birth, phone number, and email address) will be collected, this information will be stored separately to the outcome data and will be linkable only by the RA. Identifying information will be used for patient contact and linking with ORIGINS records.

### Analytic Plan

#### Quantitative Analysis

The baseline characteristics of the participants will be reported to describe the sample. Descriptive statistics will be used to summarize the quantitative data collected for each feasibility domain. Feasibility metrics will address the ADePT (*a process for decision making after pilot and feasibility trials*) framework [79]. This feasibility framework has been incorporated as part of a phased approach to the development and evaluation of the intervention. Feasibility assessment will use both quantitative feasibility data and qualitative data derived from the postprogram interviews. According to the ADePT framework, the progression criteria to move from a pilot to full-scale trial are divided into 3 categories: green, red, and amber (Table 5). Although the purpose of the study is not hypothesis testing, we will calculate the differences between the experimental groups and the control condition for primary and secondary outcomes.

**Table 5:** Decision-making criteria for full-scale randomized controlled trial, adapted from the “a process for decision making after pilot and feasibility trials” framework.

Outcome	Metrics
Green (proceed to full-scale RCT^a^ without refinement)	≥50% of eligible participants consent to the pilot trial ≥70% of consented participants commence each intervention arm ≥50% of consented participants complete all intervention sessions in each intervention arm Program satisfaction ratings and postprogram interventions demonstrate high satisfaction and acceptability of the program for ≥50% participants
Red (do not proceed to full-scale RCT)	<30% of eligible participants consent to the pilot trial <20% of consented participants commence each intervention arm Program satisfaction ratings and postprogram interventions demonstrate low satisfaction or lack of acceptability of the program for ≥50% participants
Amber (consider proceeding to full-scale trial only after protocols have been refined)	Neither red nor green criteria were met. Findings from qualitative and quantitative data will be used to determine whether a full-scale RCT should proceed with revisions

^a^RCT: randomized controlled trial.

#### Qualitative Analysis

Qualitative data collected during interviews will be analyzed to identify key themes regarding program engagement, acceptable and perceived impacts, and confidence in sustaining meditation practices. Using the approach by Braun and Clarke [80] to thematic analysis, transcripts will be iteratively coded, and codes will be collated into higher-level themes [80]. Transcripts will be reviewed to identify all instances of thematic codes, with codes expanded or collapsed as required. Qualitative data will be analyzed using qualitative content analysis [81], assigning a code to each concept using NVivo. Similar concepts will be identified and categorized into categories. Data will be analyzed using a phenomenological approach (ie, as a description of experiences as consciously experienced by participants), and narrative themes will be deducted until saturation. The coding process will be guided by the following deductive constructs: intervention acceptability, engagement, and application; mental health and well-being experiences; and suggestions for program revision.

### Data Monitoring and Harms

Adverse events will be monitored weekly based on the self-reported data provided by participants at the weekly assessment and the data collected by the ORIGINS team. The key adverse event linked to the MMM study is deteriorating mental health; however, there is also a range of potential adverse events linked to pregnancy that are recorded by ORIGINS. In the MMM study, participants will be encouraged to contact the RA at any point if they have concerns about deteriorating mental health. They are asked weekly if they wish to continue with the program. If they select “no,” they are asked about their reasons for discontinuation (including concerns about their mental or physical health). If participants report concerns about their mental health during the intervention or upon withdrawal from the study, they will be screened for depressive symptoms using the EPDS and for symptoms of traumatic stress using the abbreviated version of the Posttraumatic Stress Disorder Checklist–Civilian version (PCL-C) [82]. Participants who endorse item 10 on the EPDS or score 14 or more on the abbreviated PCL-C will receive a follow-up phone call from the study psychologist to discuss referral to more intensive or specialized support services. If a participant reports concerns about their mental or physical health, this will be reported to ORIGINS. ORIGINS has standard operating procedures for following up adverse events [62]. All adverse events in the MMM study will be recorded on an adverse event monitoring form, and all events and follow-up will be reported to an independent study monitor.

## Results

This study was funded in September 2019 and received ethics approval on July 8, 2020. Enrollment to the study will commence in September 2020. A period of 1 year is scheduled for data collection, analysis, and publication of results. The results will inform the design of an adequately powered RCT.

## Discussion

There is a need for scalable, flexible, and accessible interventions to promote the mental health of women during the perinatal period. Such interventions play an important role in supporting maternal resilience across pregnancy, potentially preventing the development of mental health problems and adjustment difficulties in the postpartum period. Although there is evidence to support the use of MBIs for improving psychological health in the perinatal period, data on the feasibility and acceptability of web-based MBIs are limited. Furthermore, little is known about the comparative benefits of alternative meditation training programs such as those that aim to cultivate loving-kindness and compassion. As no prior studies have compared MBIs and CBIs in the perinatal period, this pilot study will provide important insights into how women might differentially experience these interventions. It will also provide data to support the design of a large-scale RCT.

### Strengths

The proposed study has both strengths and limitations. The relative strength of the intervention approach is the involvement of mothers in the development of the intervention conditions. To our knowledge, there is limited literature documenting consumer involvement in the development of meditation-based interventions or in perinatal mental health promotion programs. Furthermore, the proposed trial will collect multiple measures of intervention engagement, experience, acceptability, adherence, perceived benefits, and harm. This comprehensive approach aligns with the recommendations made by Dimidjian and Segal [83] for progressing the clinical science of MBIs. In addition, the study is nested within a large longitudinal birth cohort, enabling future follow-up of study participants and their offspring. Finally, we have prespecified criteria for determining the feasibility of the study and determining whether to proceed with the full-scale RCT. Should the study proceed to a full-scale RCT, this will be one of few randomized trials of MBIs with pregnant women that use a well-matched active control condition.

### Limitations

The situation of this study within a larger longitudinal birth cohort study also has limitations, namely that participants in the study are limited to those who have already consented to participate in a research project with a significant time investment. Accordingly, these women may not be representative of the general population, and insights into the accessibility and scalability of the MMM intervention are thus limited to this group. We propose to address this in the future by conducting consultation with women and service providers at other maternity hospitals not participating in ORIGINS and to study the effectiveness and implementation of the MMM study in women giving birth in these hospitals. A further limitation of the study is the potential confounding due to the capacity of participants in the study to seek psychological treatment while participating. To explore the impact of concurrent treatment, we will measure this at baseline and posttest, with the intention of analyzing these data in a full-scale RCT. Furthermore, it is anticipated that our sample will be heterogeneous with regard to the levels of psychological distress that participants experience and types of other support accessed during the study period. Despite these limitations, we believe that the proposed trial will provide important insights into the potential utility of web-based meditation-based training as a scalable public health intervention for promoting perinatal mental health.

## Appendix

Multimedia Appendix 1 Description of mindfulness and loving-kindness and compassion practices used in the Mums Minds Matter program.

## Abbreviations

ADePT: A process for Decision making after Pilot and Feasibility Trials
CBI: compassion-based intervention
EPDS: Edinburgh Postnatal Depression Scale
JHC: Joondalup Health Campus
LKCT: loving-kindness and compassion training
MBI: mindfulness-based intervention
MMM: Mums’ Mind Matter
PMR: Progressive Muscle Relaxation
RA: research assistant
RCT: randomized controlled trial

## References

[1] Cornsweet BC, Steadman J (2017). Distress levels in pregnancy and matched non-pregnant women. ANZJOG.

[2] Arifin SR, Cheyne H, Maxwell M (2018). Review of the prevalence of postnatal depression across cultures. AIMS Public Health.

[3] Stone SL, Diop H, Declercq E, Cabral HJ, Fox MP, Wise LA (2015). Stressful events during pregnancy and postpartum depressive symptoms. J Womens Health (Larchmt).

[4] Salm Ward T, Kanu FA, Robb SW (2017). Prevalence of stressful life events during pregnancy and its association with postpartum depressive symptoms. Arch Womens Ment Health.

[5] Stein A, Pearson RM, Goodman SH, Rapa E, Rahman A, McCallum M, Howard LM, Pariante CM (2014). Effects of perinatal mental disorders on the fetus and child. Lancet.

[6] Dunkel Schetter Christine (2011). Psychological science on pregnancy: stress processes, biopsychosocial models, and emerging research issues. Annu Rev Psychol.

[7] Henrichs J, Schenk JJ, Roza SJ, van den Berg MP, Schmidt HG, Steegers EAP, Hofman A, Jaddoe VW, Verhulst FC, Tiemeier H (2010). Maternal psychological distress and fetal growth trajectories: the Generation R Study. Psychol Med.

[8] Osborne S, Biaggi A, Chua T, Du Preez A, Hazelgrove K, Nikkheslat N, Previti G, Zunszain P, Conroy S, Pariante C (2020). Corrigendum to "Antenatal depression programs cortisol stress reactivity in offspring through increased maternal inflammation and cortisol in pregnancy: The Psychiatry Research and Motherhood - Depression (PRAM-D) study" [Psychoneuroendocrinology 98 (2018) 211-221]. Psychoneuroendocrinology.

[9] van den Bergh Bea R H, van den Heuvel MI, Lahti M, Braeken M, de Rooij SR, Entringer S, Hoyer D, Roseboom T, Räikkönen Katri, King S, Schwab M (2017). Prenatal developmental origins of behavior and mental health: The influence of maternal stress in pregnancy. Neurosci Biobehav Rev.

[10] Lahti M, Savolainen K, Tuovinen S, Pesonen A, Lahti J, Heinonen K, Hämäläinen Esa, Laivuori H, Villa PM, Reynolds RM, Kajantie E, Räikkönen Katri (2017). Maternal Depressive Symptoms During and After Pregnancy and Psychiatric Problems in Children. J Am Acad Child Adolesc Psychiatry.

[11] Wolford E, Lahti M, Tuovinen S, Lahti J, Lipsanen J, Savolainen K, Heinonen K, Hämäläinen Esa, Kajantie E, Pesonen A, Villa PM, Laivuori H, Reynolds RM, Räikkönen Katri (2017). Maternal depressive symptoms during and after pregnancy are associated with attention-deficit/hyperactivity disorder symptoms in their 3- to 6-year-old children. PLoS One.

[12] Van Batenburg-Eddes T, Brion MJ, Henrichs J, Jaddoe VW, Hofman A, Verhulst FC, Lawlor DA, Davey Smith G, Tiemeier H (2013). Parental depressive and anxiety symptoms during pregnancy and attention problems in children: a cross-cohort consistency study. J Child Psychol Psychiatry.

[13] O'Donnell KJ, Glover V, Barker ED, O'Connor TG (2014). The persisting effect of maternal mood in pregnancy on childhood psychopathology. Dev Psychopathol.

[14] El Marroun H, Tiemeier H, Muetzel RL, Thijssen S, van der Knaap NJ, Jaddoe VW, Fernández Guillén, Verhulst FC, White TJ (2016). Prenatal Exposure to Maternal and Paternal Depressive Symptoms and Brain Morphology: A Population-based Prospective Neuroimaging Study in Young Children. Depress Anxiety.

[15] Wadhwa P, Buss C, Entringer S, Swanson J (2009). Developmental origins of health and disease: brief history of the approach and current focus on epigenetic mechanisms. Semin Reprod Med.

[16] Moe V, von Soest T, Fredriksen E, Olafsen KS, Smith L (2018). The Multiple Determinants of Maternal Parenting Stress 12 Months After Birth: The Contribution of Antenatal Attachment Style, Adverse Childhood Experiences, and Infant Temperament. Front Psychol.

[17] Giallo R, Cooklin A, Wade C, D'Esposito F, Nicholson JM (2014). Maternal postnatal mental health and later emotional-behavioural development of children: the mediating role of parenting behaviour. Child Care Health Dev.

[18] Da Costa D, Zelkowitz P, Nguyen T, Deville-Stoetzel J (2018). Mental health help-seeking patterns and perceived barriers for care among nulliparous pregnant women. Arch Womens Ment Health.

[19] Button S, Thornton A, Lee S, Shakespeare J, Ayers S (2017). Seeking help for perinatal psychological distress: a meta-synthesis of women's experiences. Br J Gen Pract.

[20] Woolhouse H, Brown S, Krastev A, Perlen S, Gunn J (2009). Seeking help for anxiety and depression after childbirth: results of the Maternal Health Study. Arch Womens Ment Health.

[21] Goodman JH (2009). Women's attitudes, preferences, and perceived barriers to treatment for perinatal depression. Birth.

[22] Lamers SM, Westerhof GJ, Glas CA, Bohlmeijer ET (2015). The bidirectional relation between positive mental health and psychopathology in a longitudinal representative panel study. J Posit Psychol.

[23] Grant F, Guille C, Sen S (2013). Well-being and the risk of depression under stress. PLoS One.

[24] Schotanus-Dijkstra M, Keyes CL, de Graaf R, Ten Have M (2019). Recovery from mood and anxiety disorders: The influence of positive mental health. J Affect Disord.

[25] Keyes CL, Dhingra SS, Simoes EJ (2010). Change in level of positive mental health as a predictor of future risk of mental illness. Am J Public Health.

[26] Bos SC, Macedo A, Marques M, Pereira AT, Maia BR, Soares MJ, Valente J, Gomes AA, Azevedo MH (2013). Is positive affect in pregnancy protective of postpartum depression?. Braz J Psychiatry.

[27] Robakis TK, Williams KE, Crowe S, Kenna H, Gannon J, Rasgon NL (2015). Optimistic outlook regarding maternity protects against depressive symptoms postpartum. Arch Womens Ment Health.

[28] Voellmin A, Entringer S, Moog N, Wadhwa PD, Buss C (2013). Maternal positive affect over the course of pregnancy is associated with the length of gestation and reduced risk of preterm delivery. J Psychosom Res.

[29] Huppert FA, Whittington JE (2003). Evidence for the independence of positive and negative well-being: implications for quality of life assessment. Br J Health Psychol.

[30] Slade M (2010). Mental illness and well-being: the central importance of positive psychology and recovery approaches. BMC Health Serv Res.

[31] Spijkerman M, Pots W, Bohlmeijer E (2016). Effectiveness of online mindfulness-based interventions in improving mental health: A review and meta-analysis of randomised controlled trials. Clin Psychol Rev.

[32] Galla BM, O'Reilly GA, Kitil MJ, Smalley SL, Black DS (2015). Community-Based Mindfulness Program for Disease Prevention and Health Promotion: Targeting Stress Reduction. Am J Health Promot.

[33] de Abreu Costa M, D’Alò de Oliveira GS, Tatton-Ramos T, Manfro GG, Salum GA (2018). Anxiety and stress-related disorders and mindfulness-based interventions: a systematic review and multilevel meta-analysis and meta-regression of multiple outcomes. Mindfulness.

[34] Geschwind N, Peeters F, Drukker M, van Os J, Wichers M (2011). Mindfulness training increases momentary positive emotions and reward experience in adults vulnerable to depression: a randomized controlled trial. J Consult Clin Psychol.

[35] Kirby JN, Tellegen CL, Steindl SR (2017). A Meta-Analysis of Compassion-Based Interventions: Current State of Knowledge and Future Directions. Behav Ther.

[36] Kang Y, Gray JR, Dovidio JF (2014). The Head and the Heart: Effects of Understanding and Experiencing Lovingkindness on Attitudes Toward the Self and Others. Mindfulness.

[37] Trautwein F, Kanske P, Böckler A, Singer T (2020). Differential benefits of mental training types for attention, compassion, and theory of mind. Cognition.

[38] Fredrickson BL, Boulton AJ, Firestine AM, van Cappellen P, Algoe SB, Brantley MM, Kim SL, Brantley J, Salzberg S (2017). Positive emotion correlates of meditation practice: a comparison of mindfulness meditation and loving-kindness meditation. Mindfulness (N Y).

[39] Zeng X, Chiu CP, Wang R, Oei TP, Leung FY (2015). The effect of loving-kindness meditation on positive emotions: a meta-analytic review. Front Psychol.

[40] Hofmann SG, Grossman P, Hinton DE (2011). Loving-kindness and compassion meditation: potential for psychological interventions. Clin Psychol Rev.

[41] Graser J, Stangier U (2018). Compassion and loving-kindness meditation: an overview and prospects for the application in clinical samples. Harv Rev Psychiatry.

[42] Le Nguyen KD, Lin J, Algoe SB, Brantley MM, Kim SL, Brantley J, Salzberg S, Fredrickson BL (2019). Loving-kindness meditation slows biological aging in novices: Evidence from a 12-week randomized controlled trial. Psychoneuroendocrinology.

[43] Nyklíček I, Truijens SE, Spek V, Pop VJ (2018). Mindfulness skills during pregnancy: prospective associations with mother's mood and neonatal birth weight. J Psychosom Res.

[44] van den Heuvel M, Johannes M, Henrichs J, van den Bergh B (2015). Maternal mindfulness during pregnancy and infant socio-emotional development and temperament: the mediating role of maternal anxiety. Early Hum Dev.

[45] Braeken MA, Jones A, Otte RA, Nyklíček I, Van den Bergh BR (2017). Potential benefits of mindfulness during pregnancy on maternal autonomic nervous system function and infant development. Psychophysiology.

[46] Hall HG, Beattie J, Lau R, East C, Anne Biro M (2016). Mindfulness and perinatal mental health: a systematic review. Women Birth.

[47] Taylor BL, Cavanagh K, Strauss C (2016). The effectiveness of mindfulness-based interventions in the perinatal period: a systematic review and meta-analysis. PLoS One.

[48] Shi Z, MacBeth A (2017). The effectiveness of mindfulness-based interventions on maternal perinatal mental health outcomes: a systematic review. Mindfulness (N Y).

[49] Townshend K, Caltabiano N (2019). Self-compassion and mindfulness: modeling change processes associated with the reduction of perinatal depression. J Child Fam Stud.

[50] Monteiro F, Fonseca A, Pereira M, Alves S, Canavarro MC (2019). What protects at-risk postpartum women from developing depressive and anxiety symptoms? The role of acceptance-focused processes and self-compassion. J Affect Disord.

[51] Pedro L, Branquinho M, Canavarro MC, Fonseca A (2019). Self-criticism, negative automatic thoughts and postpartum depressive symptoms: the buffering effect of self-compassion. J Reprod Infant Psychol.

[52] Kelman AR, Evare BS, Barrera AZ, Muñoz RF, Gilbert P (2018). A proof-of-concept pilot randomized comparative trial of brief Internet-based compassionate mind training and cognitive-behavioral therapy for perinatal and intending to become pregnant women. Clin Psychol Psychother.

[53] Kim MJ, Heo JM, Gim WS (2017). An Examination of the Possibility of Loving-Kindness and Compassion Meditation for Pregnant Women: A Preliminary Study. Korean J Str Res.

[54] Lippmann M, Laudel H, Heinzle M, Narciss S (2019). Relating instructional design components to the effectiveness of internet-based mindfulness interventions: a critical interpretive synthesis. J Med Internet Res.

[55] Bennett GG, Glasgow RE (2009). The delivery of public health interventions via the internet: actualizing their potential. Annu Rev Public Health.

[56] Cavanagh K, Strauss C, Forder L, Jones F (2014). Can mindfulness and acceptance be learnt by self-help?: a systematic review and meta-analysis of mindfulness and acceptance-based self-help interventions. Clin Psychol Rev.

[57] Krieger T, Reber F, von Glutz B, Urech A, Moser CT, Schulz A, Berger T (2019). An internet-based compassion-focused intervention for increased self-criticism: a randomized controlled trial. Behav Ther.

[58] Ashford MT, Olander EK, Ayers S (2016). Computer- or web-based interventions for perinatal mental health: a systematic review. J Affect Disord.

[59] Krusche A, Dymond M, Murphy SE, Crane C (2018). Mindfulness for pregnancy: a randomised controlled study of online mindfulness during pregnancy. Midwifery.

[60] Hilgart MM, Ritterband LM, Thorndike FP, Kinzie MB (2012). Using instructional design process to improve design and development of internet interventions. J Med Internet Res.

[61] Beddoe AE, Lee KA (2008). Mind-body interventions during pregnancy. J Obstet Gynecol Neonatal Nurs.

[62] Hagemann E, Colvin L, Gibson LY, Miller SJ, Palmer DJ, Srinivas Jois R, Silva DT, Prescott SL, Sata F, Fukuoka H, Hanson M (2019). The ORIGINS Project. Pre-emptive Medicine: Public Health Aspects of Developmental Origins of Health and Disease.

[63] Whitehead AL, Julious SA, Cooper CL, Campbell MJ (2016). Estimating the sample size for a pilot randomised trial to minimise the overall trial sample size for the external pilot and main trial for a continuous outcome variable. Stat Methods Med Res.

[64] Milgrom J, Ericksen J, Negri L, Gemmill AW (2005). Screening for postnatal depression in routine primary care: properties of the Edinburgh postnatal depression scale in an Australian sample. Aust N Z J Psychiatry.

[65] Jacobson E (1938). Progressive Relaxation.

[66] Lindsay EK, Chin B, Greco CM, Young S, Brown KW, Wright AG, Smyth JM, Burkett D, Creswell JD (2018). How mindfulness training promotes positive emotions: dismantling acceptance skills training in two randomized controlled trials. J Pers Soc Psychol.

[67] Dahl CJ, Lutz A, Davidson RJ (2016). Cognitive processes are central in compassion meditation. Trends Cogn Sci.

[68] Dahl CJ, Lutz A, Davidson RJ (2015). Reconstructing and deconstructing the self: cognitive mechanisms in meditation practice. Trends Cogn Sci.

[69] Engen HG, Singer T (2016). Affect and motivation are critical in constructive meditation. Trends Cogn Sci.

[70] Keyes CL (2002). The mental health continuum: from languishing to flourishing in life. J Health Soc Behav.

[71] Cox JL, Holden JM, Sagovsky R (1987). Detection of postnatal depression. Development of the 10-item Edinburgh postnatal depression scale. Br J Psychiatry.

[72] Spitzer RL, Kroenke K, Williams JB, Löwe B (2006). A brief measure for assessing generalized anxiety disorder: the GAD-7. Arch Intern Med.

[73] Cohen S, Spacapan S, Oskamp S (1988). Perceived stress in a probability sample of the United States. The Social Psychology of Health: The Claremont Symposium on Applied Social.

[74] Raes F, Pommier E, Neff KD, Van Gucht D (2011). Construction and factorial validation of a short form of the self-compassion scale. Clin Psychol Psychother.

[75] Brown KW, Ryan RM (2003). The benefits of being present: mindfulness and its role in psychological well-being. J Pers Soc Psychol.

[76] Bjureberg J, Ljótsson B, Tull MT, Hedman E, Sahlin H, Lundh L, Bjärehed J, DiLillo D, Messman-Moore T, Gumpert CH, Gratz KL (2016). Development and validation of a brief version of the difficulties in emotion regulation scale: the DERS-16. J Psychopathol Behav Assess.

[77] Thompson ER (2016). Development and validation of an internationally reliable short-form of the positive and negative affect schedule (PANAS). J Cross-Cult Psychol.

[78] Herdman M, Gudex C, Lloyd A, Janssen M, Kind P, Parkin D, Bonsel G, Badia X (2011). Development and preliminary testing of the new five-level version of EQ-5D (EQ-5D-5L). Qual Life Res.

[79] Bugge C, Williams B, Hagen S, Logan J, Glazener C, Pringle S, Sinclair L (2013). A process for decision-making after pilot and feasibility trials (ADePT): development following a feasibility study of a complex intervention for pelvic organ prolapse. Trials.

[80] Braun V, Clarke V (2006). Using thematic analysis in psychology. Qual Res Psychol.

[81] Sandelowski M (2000). Whatever happened to qualitative description?. Res Nurs Health.

[82] Lang AJ, Stein MB (2005). An abbreviated PTSD checklist for use as a screening instrument in primary care. Behav Res Ther.

[83] Dimidjian S, Segal ZV (2015). Prospects for a clinical science of mindfulness-based intervention. Am Psychol.

