# Supplementary material for: Comparing Web-Based Mindfulness With Loving-Kindness and Compassion Training for Promoting Well-Being in Pregnancy: Protocol for a Three-Arm Pilot Randomized Controlled Trial
Source: JMIR Res Protoc. 2020 Oct 14;9(10):e19803. doi: 10.2196/19803 (PMC7593853; doi:10.2196/19803)
Supplement: Multimedia Appendix 1 [file resprot_v9i10e19803_app1.docx]

Appendix. Description of mindfulness and loving-kindness/compassion practices used in the Mums Minds Matter program.

| Mindfulness Practices | |
| --- | --- |
|  | |
| Body scan | In this practice, participants are guided through a process of focusing their attention on different parts of their body in turn. There is an emphasis on building interoceptive awareness, cultivating acceptance, and noticing the habits of the mind during the practice (e.g. tendency to mind-wander or judge experiences). |
| Breath focused meditation  Mindfulness of sound  Mindfulness of feelings | In the MMM program, the breath-focused meditation practice is used over three weeks, with variation to the instructions provided. In this practice, participants are guided through a process of focusing their attention on their breath as a stable – though dynamic – anchor for the mind. In this focused attention practice, participants are encouraged to notice the habits of the mind during the practice (e.g. the tendency to mind-wander or judge experiences) and bring their attention back to their breath.  In the second iteration of the practice, participants are guided through a process of balancing focused attention on the breath with awareness of sounds as they come and go.  In the third iteration of the practice week, there is an additional emphasis on allowing thoughts, feelings, and bodily sensations to come and go without judging them or getting caught up in them. |
| Walking meditation | In this practice, participants are guided through a process of focusing their breath and bodily sensations while walking. This practice helps to build interoceptive awareness while also integrating mindfulness into a ‘regular’ activity such as walking. |
| Mountain meditation | This practice focuses on supporting participants to develop equanimity, using the imagery of a mountain. Participants are guided through a process of visualizing and embodying a mountain, connecting with a sense of groundedness and calm abiding regardless of the changing weather and seasons (a metaphor for changing thoughts and feelings) that they may observe. |
| Loving-Kindness/Compassion Practices | |
| Compassionate check-in | In the practice, participants are guided through a process of ‘checking in’ with their feelings and physical sensations with curiosity and friendliness. The intention of this practice is to familiarize participants with the idea of caring about their own wellbeing in the same way they would a friend or loved one. |
| Compassionate body scan | In this practice, participants are guided through a process of focusing their attention on different parts of their body in turn. There is an emphasis on meeting difficult sensations, feelings or thoughts with gentleness and unconditional friendliness. Participants are encouraged to actively soothe and soften parts of their body where they notice tension or uncomfortable feelings. |
| Soothing image practice | In this practice participants are guided through a process of visualizing different images that give them a sense of being soothed, calmed, and connected to others. The intention of this practice is to familiarize participants with the felt sense of being soothed, as well as to build self-efficacy for soothing themselves when they are stressed or experiencing physical or emotional pain. |
| Loving-kindness for a loved one  Loving-kindness for self  Giving and receiving loving-kindness | In the MMM program, the loving-kindness practice is used over three weeks, with variation to the instructions provided. The intention of this practice is to increase feelings of connectedness with others and build familiarity with having benevolent and compassionate intentions towards oneself, one’s baby and others. The first practice guides participants through a process of bringing someone they care about (a loved one, their baby, a friend, or a pet) to mind and extending wishes of loving-kindness towards that being. They are invited to imagine their wishes bringing the other person happiness and peace.  In the second practice, participants are again asked to extend wishes of loving-kindness to someone they care about, and then to visualize extending the circle of loving-kindness so that they are also included in the wish for happiness and peace.  In the third practice, participants are asked to visualize extending the circle of loving-kindness beyond themselves and loved ones, to others that they do not know (their community, neighborhood, etcetera). They are also invited to imagine that others are directing wishes of loving-kindness back toward them. This practice is reinforced by imagining “breathing loving-kindness in and out” as they give and receive loving-kindness to and from the different groups that they bring to mind. |
| Compassionately responding to emotions | In this practice, participants are invited to recall a mild emotional difficulty or sense of physical discomfort. They are guided through a process of acknowledging the emotional and physical experience and responding to it with kindness and compassion – in the same way they would respond to a friend who was experiencing a similar difficulty. Participants are encouraged to try different strategies to respond to the difficult experience – such as softening or soothing the part of their body where they experience the emotional or physical sensation, imagining receiving care and kindness from someone (or some place) that they find soothing, or repeating words of kindness and compassion to themselves. |
